# Pathogenic Mechanism of Der p 38 as a Novel Allergen Homologous to RipA and RipB Proteins in Atopic Dermatitis

**DOI:** 10.3389/fimmu.2021.646316

**Published:** 2021-10-08

**Authors:** Hyang Jeon, Geunyeong Kim, Ayesha Kashif, Min Hwa Hong, Ji-Sook Lee, Yujin Hong, Beom Seok Park, Eun Ju Yang, In Sik Kim

**Affiliations:** ^1^ Department of Senior Healthcare, Graduate School, Eulji University, Uijeongbu, South Korea; ^2^ Department of Clinical Laboratory Science, Wonkwang Health Science University, Iksan, South Korea; ^3^ Department of Biomedical Laboratory Science, College of Health Science, Eulji University, Seongnam, South Korea; ^4^ Department of Biomedical Laboratory Science, Daegu Haany University, Gyeongsan, South Korea; ^5^ Department of Biomedical Laboratory Science, College of Health Science, Eulji University, Uijeongbu, South Korea

**Keywords:** Der p 38, atopic dermatitis, filaggrin, TLR4, skin barrier

## Abstract

Atopic dermatitis (AD) is a chronic relapsing pruritic disease encompassing skin inflammation and barrier dysfunction. House dust mites are key allergens that augment the development of atopic dermatitis. We aimed to investigate the pathogenic mechanism of AD due to Der p 38, recently identified by us. The frequency of IgE reactivity to Der p 38 in AD subjects was 52.6% (10/19) in the skin prick test and 57.9% (11/19) in the dot blot assay. In human keratinocyte HaCaT cells, Der p 38 triggered the impairment of filaggrin expression and induced pro-inflammatory cytokines such as IL-6, IL-8 and MCP-1 through TLR4, PI3K, AKT, c−Jun N−terminal kinase (JNK) and NF-κB pathway. Supernatants from Der p 38-treated cells blocked filaggrin expression and neutrophil apoptosis. The anti-apoptotic effect of the Der p 38-released molecules on neutrophils was accomplished by inhibition of the caspase 9/3 pathway, and by increased MCL-1 expression and BCL-2/BAX expression ratio. In C57BL/6 wild type (WT) mice, Der p 38 induced a dose-dependent increase of AD-like skin lesions, with enhanced expressions of total and Der p 38-specific IgE. Der p 38 also diminished the expressions of skin barrier proteins and induced JNK activation. However, the AD-like features following cutaneous Der p 38 exposure were observed to be reduced in the TLR4 knockout (KO) group, as compared to the WT group. Skin infiltration of neutrophils, eosinophils and mast cells was increased in the WT mice, but was not portrayed in the TLR4 KO mice. These findings indicate that Der p 38 is a novel mite allergen that triggers AD by lowering skin barrier proteins and increasing inflammatory cells. Results of this study have thereby paved the way to unveil the pathogenic mechanisms of AD.

## Introduction

Atopic dermatitis (AD) is a pruritic, relapsing and chronic inflammatory skin condition with variable clinical features (1). AD pathogenesis has mainly been explained by two pathogenic mechanisms: the “inside-to-outside” and “outside-to-inside” hypothesis (2, 3). In the inside-to-outside hypothesis, traditionally immunological aberrations are related to a Th2 immunity leading to increased IL-4, IL-5, IL-13 and IgE in an acute phase, subsequently progressing to chronic inflammation, thereby ascertaining the transition of Th2 to Th1 immunity. Another hypothesis assumes the importance of an intrinsic defect in the epidermal barrier. AD pathogenesis research has recently targeted that immune and skin barrier abnormalities contribute to the overall phenotype (4). Filaggrin disruption allows exogenous immune stimuli such as allergens to enter the skin and activate immune responses including cytokine secretion, which subsequently suppresses filaggrin expression, and vice versa (5–7). Moreover, this vicious cycle of filaggrin regulation is related to activation of c−Jun N−terminal kinase (JNK) and NF-κB (8, 9).

House dust mite (HDM) allergens play essential roles in the onset and development of AD (10–13). The major mite allergenic components, viz., protease allergens (group 1, 3) and non-protease allergens (group 2, 7), are derived from *Dermatophagoides pteronyssinus* (DP) or *Dermatophagoides farinae* (DF), and induce serum-specific IgE and Th2 immunity. TLR4 is a major receptor in HDM-induced allergic inflammation (14–17). TLR4 acts as a critical factor in severity of AD in the presence of its ligand LPS-induced infections, and this alteration of TLR4 expression is consequential in the skin of AD (18, 19). However, studies have reported that TLR4 is a negative regulator in the pathogenic mechanism of AD. Deletion of TLR4 suppresses AD-like symptoms and skin inflammation in AD-like mice due to fungus allergens and 2,4-dinitrochlorobenzene (DNCB) (20–22). Der p 2 does not induce AD *via* TLR4, but through TLR2 (23). Thus, relationship of the allergen and TLR4 with AD has remained controversial so far.

Based on the substantial available evidence that cutaneous defect of the skin barrier, allergen sensitization and TLR4 are important factors in AD pathogenesis, we examined, for the first time, the skin barrier dysfunction and TLR4-involved inflammation due to a novel allergen, Der p 38. This novel allergen was discovered by us from the DP extract, and is registered in the allergen database approved by the WHO/IUIS Allergen Nomenclature Sub-committee.

## Materials and Methods

### Reagents

DP was obtained from the Korea National Arthropods of Medical Importance Resource Bank (Yonsei University, Seoul, Korea) and Cosmo Bio (Tokyo, Japan). CLI-095, an inhibitor of Toll like receptor 4 (TLR4i), was purchased from Invitrogen (San Diego, CA, USA). PI3K inhibitor (LY294002), AKT inhibitor (AKTi), JNK inhibitor (SP600125) and NF-κB inhibitor (BAY-11-7085) were purchased from Calbiochem (San Diego, CA, USA). Antibodies against phosho-ERK1/2, MCL-1, BCL-2 and BAX were purchased from Cell Signaling Technology (Beverly, MA, USA). Antibodies against filaggrin, involucrin, loricrin, phospho-AKT, AKT, phospho-JNK, JNK, ERK2, caspase 3, and caspase 9 were obtained from Santa Cruz Biotechnology (Santa Cruz, CA, USA). Antibodies against Ly6G and tryptase were obtained from Abcam (Cambridge, UK). Antibodies against eosinophil peroxidase were obtained from Bioss Antibodies (Woburn, MA, USA) (**Supplementary Material**).

### Sequence Alignment

The protein sequences of RipB [Protein Data Bank (PDB) Code: 3PBI], RipA [PDB code: 3PBC] and Der p38 (GenBank Protein ID: QLY71953) proceeded multiple sequence alignment using Clustal Omega program (https://www.ebi.ac.uk/Tools/msa/clustalo). The aligned sequences were represented by the ESPript 3.0 program to render sequence similarities and secondary structure information (http://espript.ibcp.fr/ESPript/ESPript/index.php).

### Expression of Recombinant Der p 38 and Generation of the Antibody

cDNAs of DP were synthesized from total RNAs of DP using the iScript cDNA synthesis kit (Bio-Rad, Hercules, CA, USA). Der p 38 cDNA (GenBank accession number MT273069.1) was synthesized by PCR using forward (5’- ACT CAG GAT CCG ATG CAA GTT TAT GGT AAT GGT-3’) and reverse primers (5’- ACT ACG CGG CCG CTC ACC AAC ATC GTG CAA CAT TAG C -3’), and the amplified cDNA was inserted into the plasmid pETDuet-1(Merck Millipore, Darmstadt, Germany). *E. coli* Orgami B (DE3) cells transformed with the pETDuet-1 expression vector were grown to an A600 nm of 0.6 before induction with 0.5 mM isopropyl β-D-1-thiogalactopyranoside (IPTG). Subsequently, the harvested cells were lysed by sonication in lysis buffer followed by purification using a nickel column (Merck Millipore). A 10 kDa Amicon Ultra concentrator (Millipore) was used for concentrating the protein, followed by purification using a Superdex 200 column attached to an ÄKTA FPLC system (GE Healthcare). The endotoxin level was determined by the Toxin sensor chromogenic LAL endotoxin assay kit (GenScript, Piscataway, NJ, USA). The mandatory removal of endotoxins was achieved by using the ToxinEraser endotoxin removal kit (GenScript). Sodium dodecyl sulfide -polyacrylamide gel electrophoresis (SDS-PAGE) was performed for identification of the protein, which was stained with Coomassie brilliant blue. Protease activity of Der p 38 was measured using the fluorescent protease assay kit (Pierce, Rockford, IL, USA). The purified protein was aliquoted and stored at −70°C for further use. Polyclonal antibodies against Der p 38 were produced by immunizing rabbits. After first injection of the purified Der p 38, rabbits were boosted by the same protein two times. Enzyme-linked immunosorbent assay (ELISA) was performed as activity test of the antibodies after coating with Der p 38 protein. Antibodies at diluted sera (1:1000, 1:5000, 1:10000, and 1:50000) showed O.D. greater than (>) 1.0.

### Subjects

Healthy subjects and AD volunteers with no medication were recruited at the Eulji University for blood donation and undergoing skin prick test (**Table 1** and **Supplementary Table 1**). The human blood protocol and associated consent forms were reviewed and approved by the Institutional Review Board (IRB) of the Eulji University. Conventional skin prick test was conducted according to the instructions of the SoluprickR test. Histamine (positive control), DP, DF (ALK Horsholm, Denmark), Der p 1, Der p 2 (INDOOR Biotechnologies, Charlottesville, VA, USA) and Der p 38 were used as test materials. Recombinant allergen proteins (10 μg/mL) were solubilized in phosphate buffered saline (PBS, pH 7.4) before using in this test. A positive result was determined as a ≥1+ (i.e., a wheal ≥3 mm diameter).

**Table 1:** Demographic and clinical characteristics of normal and AD subjects.

	Normal (n=19)		AD (n=19)	
Number of subjects (female/male)	19 (15/4)		19 (15/4) AD (12), AD+AR (5), AD+AS (2)	
Age (years)	24.9 ± 5.2 (20~41)		23.5 ± 6.0 (20~48)	
Total IgE (ng/mL)	158.6 ± 171.7 (38.9~603.9)		361.3 ± 457.9 (47.8~1594.5)*	
DP+, DF+	5 (26.3%)		17 (89.5%)	
DP+, DF-	1 (5.3%)		2 (10.5%)	
DP-, DF+	0 (0%)		0 (0.0%)	
DP-, DF-	13 (68.4%)		0 (0.0%)	
Der p 1	2(10.5%)		14 (73.7%)	
Der p 2	3 (15.8%)		12 (63.2%)	
Der p 38	3 (15.8%)		10 (52.6%)	
	HDM + (n=6)	HDM – (n=13)	HDM + (n=19)	HDM – (n=0)
Der p 1	2 (33.3%)	0 (0.0%)	14 (73.7%)	0 (0.0%)
Der p 2	3 (50.0%)	0 (0.0%)	12 (66.2%)	0 (0.0%)
Der p 38	3 (50.0%)	0 (0.0%)	10 (52.6%)	0 (0.0%)

AD, atopic dermatitis; AR, atopic rhinitis; AS, asthma.

*Significant difference (p<0.05) between normal and AD subjects.

A positive result in the skin prick test is determined as a ≥1+ (i.e., a wheal ≥3 mm diameter).

HDM+ is described as at least one of DP and DF is + in the skin prick test.

### IgE Reactivity to Der p 38

IgE reactivity was determined by the dot assay. Briefly, recombinant Der p 38 proteins were added to nitrocellulose membrane and dried. The membrane was blocked with 3% bovine serum albumin (BSA) solution for 30 min, and then incubated with diluted (1:10) sera of healthy or AD subjects for 1 h. After incubating with secondary mouse anti-human IgE antibody (Abcam, Cambridge, UK), the membrane was developed using the enhanced chemiluminescence detection system (Thermo Scientific, Waltham, MA, USA).

To measure the concentration of IgE against Der p 38 protein in the sera of mice, plates were coated with Der p 38 protein at a concentration of 1 μg/mL per well, in carbonate buffered solution at 4°C overnight. For preventing non-specific reaction, 5% BSA solution was added to each well. After washing three times, the plate was incubated with sera (1:5), followed by incubation with biotin-labeled goat anti-mouse IgE and SAv-HRP reagent added to each well. For stopping the reaction, 2M H_2_SO_4_ solution was added to each well, and absorbance was measured at 450 nm using an automated microplate reader (Molecular Devices, Chicago, IL, USA).

### Total IgE and Cytokine ELISA

The human IgE kit (Carlsbad, CA, USA) and mouse IgE ELISA set (BD Biosciences, San Diego, CA, USA) were used to evaluate the total IgE in the sera of human and mice, respectively, according to the recommended protocols. To measure cytokine concentration, HaCaT cells were stimulated with Der p 38 in a time-dependent manner, and supernatants were collected. OptEIA Set human IL-6, IL-8 and MCP-1 (BD Biosciences), were used for estimating concentrations of IL-6, IL-8 and MCP-1, respectively, in the supernatants, according to the manufacturer’s guidelines. Absorbance was measured at 450-550 nm using the automated microplate reader procured from Molecular Devices (San Jose, CA, USA).

### Cell Culture

The human keratinocyte cell line (HaCaT) was maintained in Dulbecco’s modified Eagle’s medium supplemented with 10% FBS and penicillin/streptomycin, under an atmosphere of 95% air/5% CO_2_ at 37°C.

### Western Blotting and NF-κB p65 Transcription Factor Assay

HaCaT cells were treated with 10 μg/mL Der p 38 for the indicated time, and then harvested cells were lysed in lysis buffer (TransLab, Daejeon, Korea). The homogenate was centrifugated at 12,000 x g for 15 min at 4°C, and the supernatant was collected as total lysate. Protein concentration of the lysate was measured by a protein assay kit (Thermo Scientific). Following separation of the protein samples (50 μg/lane) by 10% SDS-PAGE, the transferred nitrocellulose membrane was sequentially incubated with primary (1:1,000) and secondary antibody (1:3,000) for 1 h at room temperature, and developed using the Enhanced Chemiluminescence Western blotting Detection System (Amersham Pharmacia Biotech, Piscataway, NJ, USA). The same blot was stripped and re-probed with internal control antibodies, such as anti-ERK2 antibodies. For evaluating NF-κB activity, nuclear lysates were assessed for NF-kB DNA-binding activity using NF-κB p65 transcription factor assay kit (Abcam, Cambridge, UK) as described in our previous paper (24).

### Separation of Neutrophils and Eosinophils

Heparinized peripheral blood was collected from healthy and AD subjects, and layered on Ficoll-Hypaque gradient solution. After centrifugation, the leukocytes were collected, and the erythrocyte contaminants were eliminated by RBC lysis buffer. Both neutrophils (CD16+) and eosinophils (CD16-) were separated by applying the CD16 microbeads magnetic cell sorting kit (Miltenyi Biotec, Bergisch Gladbach, Germany). Isolated cells were washed three times by PBS buffer and resuspended in RPMI 1640 medium containing 10% fetal bovine serum (FBS) and antibiotics. Purity of the cells was determined to be greater than 97%, as evaluated by cell counting after subjecting to Wright-Giemsa stain.

### Detection of Apoptosis

Neutrophils and eosinophils were incubated with FITC-labeled annexin V and propidium iodide (PI) (BD Biosciences, San Diego, CA, USA) for 15 min at room temperature. The stained cells were analyzed by RF-500 (Sysmex Corporation, Kobe, Japan) and were divided into early apoptotic cells (Annexin V+, PI-), late apoptotic cells or necrotic cells (Annexin V +, PI +) and viable cells (Annexin V-, PI-). Early and late apoptotic cells are considered apoptotic cells. The percentage of cells showing annexin V+/PI- and annexin V+/PI+ cells were considered as apoptotic neutrophils and eosinophils.

### AD Induction Subsequent to Der p 38 Administration

C57BL/10ScNJ and C57BL/10ScNJ TLR4 knockout (KO) mice were kindly provided by Jeong Won-Il (Korea Advanced Institute of Science and Technology; KAIST). All animals were maintained in a specific pathogen-free (SPF) facility. All the animal experiments were performed in accordance with the protocols approved by the Institutional Animal Care and User Committee of Eulji University. WT mice were randomly divided into five groups (n=5): control (Con), DP (100 μg per dose), and Der p 38 treatment (20, 50, and 100 μg per dose) groups. TLR4 KO mice were classified into three groups: control (Con) and Der p 38 treatment (20 and 50 μg per dose) groups. The mice were allowed to adapt for 1 week before experimental procedures. A day before the first allergen exposure, the mice backs were shaved with an electric razor. The stimulators suspended in PBS or PBS (control), were applied to a 2 x 2 cm patch of sterile gauze. The patch was secured by wrapping the mice with a band aid. After 6 days, the patch was removed, and 24 h later a new patch was applied. This continued for a total of five patches over the 5 weeks period. The clinical skin severity score was evaluated using the modified scoring atopic dermatitis (SCORAD) index. A score of 0-3 (0, none; 1, mild; 2, moderate; 3, severe) was assigned for each of the following five symptoms: pruritus/itching, erythema/hemorrhage, scarring/dryness, excoriation/erosion, and edema.

### Histological Analysis

Subsequent to sacrifice of the mice by CO_2_ asphyxiation, the dorsal skin was removed, fixed in Carnoy’s solution (Sigma-Aldrich Korea, Seoul, Korea), embedded in paraffin and sectioned (3 μm thick slices) using a microtome (Leica, Nussloch, Germany). The sections were stained with hematoxylin-eosin solution (Sigma-Aldrich Korea). Thickness of the epidermis and dermis were evaluated as described in a previous paper (23). For immunohistochemical staining for filaggrin, loricrin, involucrin and Ly6G, eosinophil peroxidase, and tryptase, sections were deparaffinized by xylene. A solution of proteinase K was applied for 30 min for antigen retrieval, followed by treatment with 0.3% H_2_O_2_ in methanol for 40 min. Slides were blocked by blocking buffer for 1.5 h, and subsequently incubated for 90 min (filaggrin) or overnight (loricrin, involucrin, Ly6G, eosinophil peroxidase, and tryptase) at 4°C with the indicated primary antibodies. After washing, the slides were incubated with secondary antibodies for 60 min (loricrin and involucrin) or 80 min (filaggrin) at room temperature. After incubating with AB and 3,3′-diaminobenzidine (DAB reagents), the specimens were counterstained with hematoxylin and examined under light microscopy (Leica Microsystems, Wetzlar, Germany) for histological evaluation. Vectastain elite ABC HRP kit (Vector Labs, Burlingame, CA, USA) and DAB chromogen were used for detecting antibodies.

### Statistical Analysis

Data are presented as the means ± SD. Statistical intergroup differences were analyzed using a paired t-test for a two-group comparison and one-way ANOVA for more than two groups. The SPSS statistical software package (Chicago, IL, USA) was used for statistical evaluation. A *p* value < 0.05 is considered statistically significant.

## Results

### Der p 38 Has IgE Reactivity in AD Subjects

**Figure 1:**
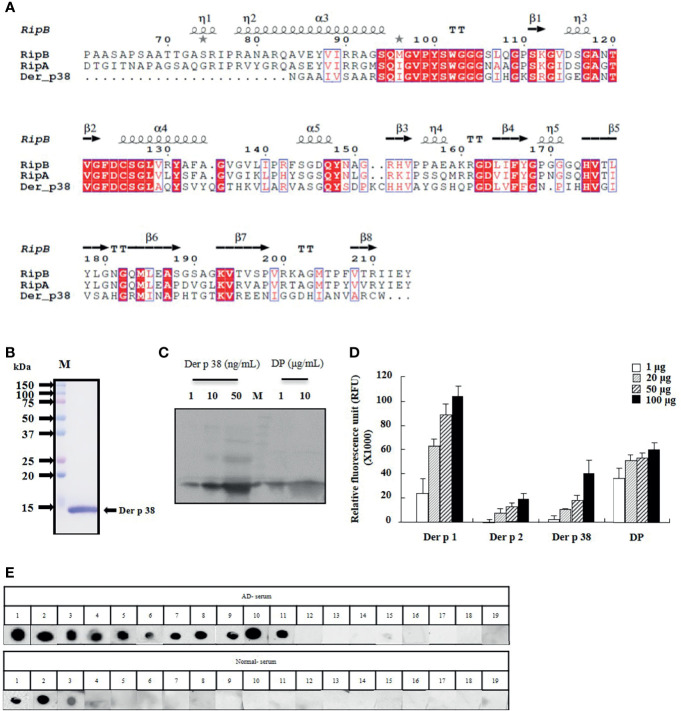
Der p 38 shows IgE reactivity in AD subjects. **(A)** Der p 38 amino acid sequence is aligned with the RipA and RipB proteins. Red box (strict identity). Blue frame (similarity across groups). Red character (similarity in a group). **(B)** Der p 38 cDNA were amplified by PCR using gene specific primer. The cDNA was cloned to the vector and transformed into *E coli*. Recombinant Der p 38 protein was produced, separated by SDS-PAGE, and stained with Coomassie brilliant blue. **(C)** Natural Der p 38 protein in DP extract was detected by Western blotting using antibodies against Der p 38. **(D)** Protease activity of Der p 38 was measured by a protease activity kit. **(E)** Dot blot assay was performed with sera of healthy and AD subjects.

Der p 38 was purified as a TLR4-binding protein from DP extract using a column attached with recombinant TLR4 protein, as compared to finding novel allergens with an IgE-binding frequency (25, 26). The protein sequence of Der p 38 is homologous to that of RipA and RipB proteins, and alignment of Der p 38 with these proteins is presented in **Figure 1A**. A recombinant Der p 38 protein was produced as described in the Materials and Methods section (**Figure 1B**). The interaction of recombinant Der p 38 with TLR4 was confirmed using a TLR4-bound column (**Supplementary Figure 1**). Content of Der p 38 in DP extract was approximately evaluated by comparing with the concentration of recombinant Der p 38 (**Figure 1C**). Since RipA and RipB proteins are peptidoglycan endopeptidases, we examined the proteolytic activity of Der p 38. As shown in **Figure 1D**, Der p 38 showed weak activity, which was lower than activity of Der p 1. Skin prick test was performed to investigate the exposure of Der p 38 in AD subjects and 52% patients were found to be sensitized by Der p 38 (**Table 1**). Positive reactions to Der p 1 and Der p 2 were 73.7% and 63.2% of the AD groups, respectively. In addition, Der p 38+ results were determined to be 57.9%, using the dot blot assay (**Figure 1E**).

### Der p 38 Diminishes Filaggrin Expression *via* TLR4, PI3K, AKT, JNK and NF-κB in Human Keratinocyte HaCaT Cells

**Figure 2:**
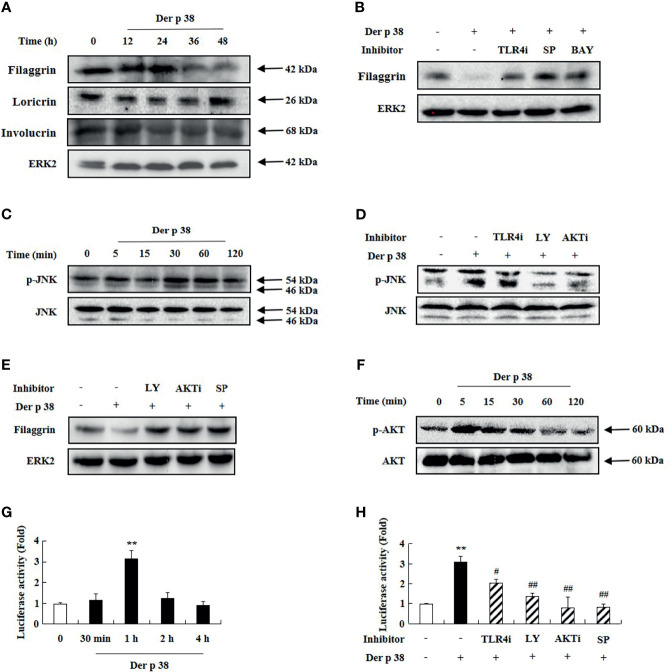
Der p 38 diminishes filaggrin expression *via* TLR4, PI3K, AKT, JNK and NF-κB in human keratinocyte HaCaT cells. **(A, C, F)** HaCaT cells were treated with 10 μg/mL Der p 38 for the indicated duration. The harvested cells were lysed, and filaggrin, loricrin, involucrin **(A)**, phospho-JNK **(C)**, and phospho-AKT **(F)** were analyzed by Western blotting. **(B, D)** The cells were pretreated with 2 μM TLR4i, 10 μM LY294002 (LY), 10 μM AKTi, 10 μM SP600125 (SP) or 10 μM BAY-11-7085 (BAY) for 1 h, following Der p 38 treatment for 48 h **(B)** or 30 min **(D)**. Filaggrin and phospho-JNK were analyzed by Western blotting. **(E)** The cells were pretreated with 10 μM LY294002, 10 μM AKTi or 10 μM SP600125, after which they were treated with Der p 38 for 48 h. Filaggrin was analyzed by Western blotting. ERK2 was used as an internal control. **(G, H)** The cells were treated with 10 μg/mL Der p 38 in a time dependent manner **(G)**, or 10 μg/mL Der p 38 after pretreatment with 2 μM TLR4i, 10 μM LY294002, 10 μM AKTi, or 10 μM SP600125 **(H)**. After lysis of the harvested cells, NF-κB activity in the lysates was evaluated by the luciferase assay. The data are presented relative to the control, which was set at 1 as the mean ± S.D (n=3). **p < 0.01 indicates a significant difference between untreated and Der p 38-treated groups, and ^#^ *p* < 0.05 and ^##^ *p* < 0.01 indicate significant difference between the Der p 38-treated group and the inhibitor-treated groups.

Since skin barrier proteins are critical elements in maintaining the skin structure and their dysfunction is related to AD aggravation, we examined whether Der p 38 affects the expression of the relevant proteins in HaCaT cells. As shown in **Figure 2A**, exposure to Der p 38 inhibits filaggrin expression and weakly downregulates the expressions of loricrin and involucrin (**Figure 2A**). Since JNK and NF-κB are important proteins in filaggrin expression (8, 9), we investigated the association of both proteins with the filaggrin downregulation mechanism after Der p 38 treatment. Inhibitors of TLR4, JNK and NF-κB reversed the inhibition of filaggrin expression exerted by Der p 38, and increased JNK activation, in a time-dependent manner (**Figures 2B, C**). To find the upstream molecules of JNK, we used specific signal inhibitors. TLR4i, LY294002 and AKTi suppressed the JNK phosphorylation and blocked the filaggrin expression inhibited by Der p 38 (**Figures 2D, E**). Furthermore, AKT activation was confirmed after treatment with Der p 38 (**Figure 2F**). NF-κB activity was time-dependently increased by Der p 38, which was suppressed by inhibitors of TLR4, PI3K, AKT and JNK (**Figures 2G, H**). Since alteration of TLR4 expression is important in regulating the agonistic effect of TLR4 (27), we investigated whether Der p 38 increases both total and surface expressions of TLR4. We observed that the total and surface TLR4 expressions remained unaltered after Der p 38 stimulation (**Supplementary Figure 2**).

### Der p 38 Increases Mediator Secretion in Human Keratinocyte HaCaT Cells, Which Prevents Filaggrin Expression and Neutrophil Apoptosis

**Figure 3:**
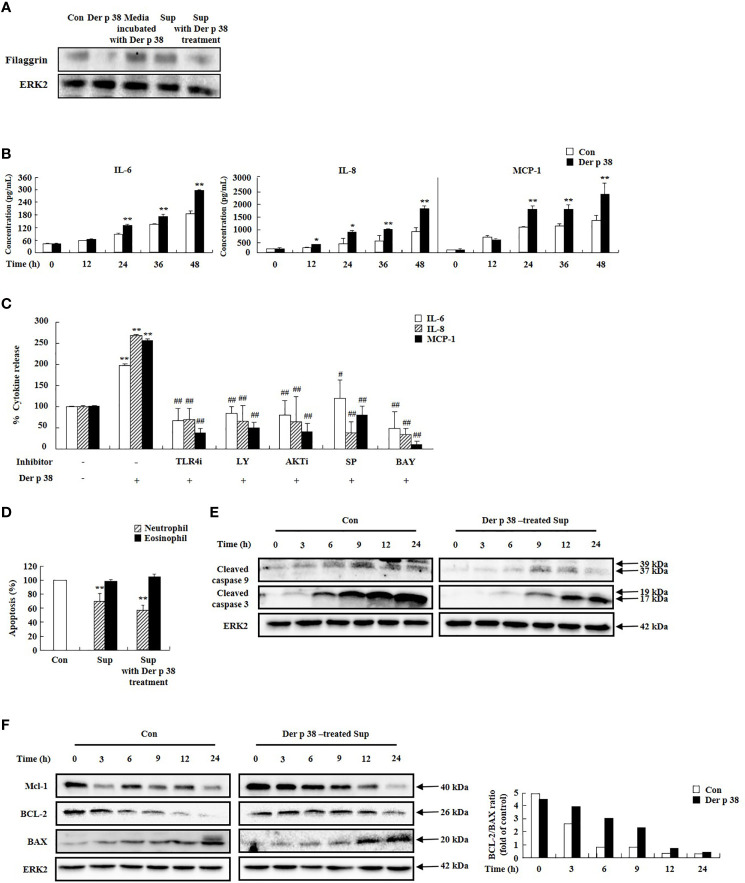
Der p 38 increases cytokine secretion in human keratinocyte HaCaT cells, thereby preventing filaggrin expression and neutrophil apoptosis. **(A)** HaCaT cells were treated with 10 μg/mL Der p 38 for 48 h. Supernatant (Sup) was collected, and the other cells were treated with the supernatant for 48 h. The harvested cells were lysed in lysis buffer and the homogenate was centrifugated, and the supernatant was collected as total lysate. Following separation of the protein samples by 10% SDS-PAGE, the transferred nitrocellulose membrane was sequentially incubated with anti-filaggrin (1:1,000) and secondary antibody (1:3,000) for 1 h at room temperature, and developed using the Enhanced Chemiluminescence Western blotting Detection System. ERK2 expression is used as an internal control. **(B, C)** Cells were treated with 10 μg/mL Der p 38 for the indicated time **(B)**, or for 48 h after pretreatment with 2 μM TLR4i, 10 μM LY294002 (LY), 10 μM AKTi, 10 μM SP600125, and 10 μM BAY-11-7085 (BAY) **(C)**. The supernatant was collected and analyzed by ELISA. **(D)** Neutrophils and eosinophils were treated with the supernatant after treatment with 10 μg/mL Der p 38. Apoptosis was analyzed by measuring the binding of annexin V-FITC and PI. The data are presented relative to the control, which was set at 100% as the mean ± S.D (n=3). *p < 0.05 and **p < 0.01 indicate a significant difference between the untreated and Der p 38-treated groups, and ^#^ *p* < 0.05 and ^##^ *p* < 0.01 indicate significant difference between the Der p 38-treated group and the inhibitor-treated groups. **(E, F)** Activation and expression of the indicated signal proteins were evaluated by Western blotting. Lower panel **(F)** is the ratio of BCL-2/BAX using densitometry.

We next investigated the relationship of the decreased filaggrin expression by Der p 38, with cytokine secretion. Filaggrin expression was suppressed by supernatant of cells treated with Der p 38 (**Figure 3A**). Since Der p 38 in the supernatant after treatment affects filaggrin expression, we evaluated the effect of media incubated with Der p 38 for 48 h; this media had no effect on filaggrin expression. To clarify the effect of the supernatant, we evaluated for levels of cytokines, such as IL-6, IL-8, and MCP-1. Exposure to Der p 38 increased these cytokine levels in a time-dependent manner (**Figure 3B**). Upregulation of IL-6, IL-8 and MCP-1 was suppressed by inhibitors of TLR4, PI3K, AKT, JNK and NF-κB (**Figure 3C**). Since these cytokines may be involved in survival of inflammatory cells, as reported in our previous papers (15, 27), we investigated alteration of granulocyte apoptosis due to Der p 38. Compared to the control supernatants, neutrophil apoptosis was inhibited by the supernatant treated with Der p 38, but eosinophil apoptosis was not altered (**Figure 3D**). The activation of caspase 9 and caspase 3 was involved in the inhibitory mechanism exerted by Der p 38-treated supernatant (**Figure 3E**). Additionally, Der p 38 increased both MCL-1 expression and the ratio of BCL-2/BAX expression, indicating that these mechanisms delay neutrophil apoptosis (**Figure 3F**).

### Der p 38 Induces AD-Like Phenotypes, Including Filaggrin Downregulation, *via* TLR4 in Mice

**Figure 4:**
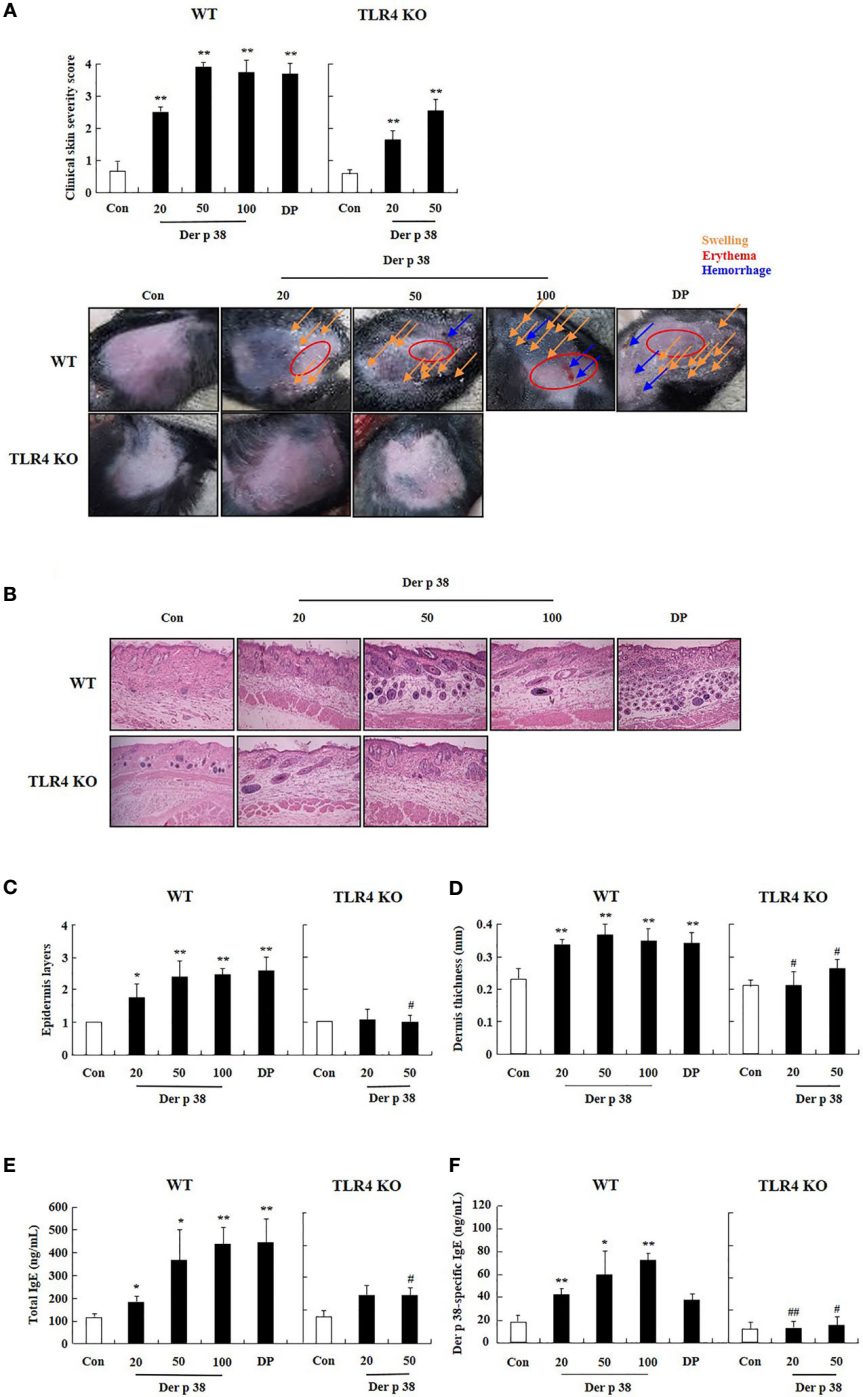
Der p 38 induces AD-like phenotypes *via* TLR4 in the mice. C57BL/10ScNJ wild type (WT) and C57BL/10ScNJ TLR4 knockout (KO) mice were used in this experiment. The mice were dorsally administered Der p 38 (20, 50, or 100 μg/mice) or DP (100 μg/mice) using a 2 x 2 cm patch of sterile gauze. The mice were wrapped with a band aid. After 6 days, the patch was removed, and 24 h later a new patch was applied, for a total of five patches over a 5 weeks period. Clinical skin severity score **(A)**, histological features using hematoxylin and eosin stain **(B)**, layers of epidermis **(C)** and dermis **(D)**, total IgE **(E)**, and Der p 38-specific IgE **(F)** were evaluated as described in the Materials and Methods section. The data are presented as a mean ± SD (n=3). *p < 0.05 and **p < 0.01 indicate a significant difference between the control group and stimulator-treated groups, and ^#^ *p* < 0.05 and ^##^ *p* < 0.01 indicate significant difference between the WT and the TLR4 KO groups.

**Figure 5:**
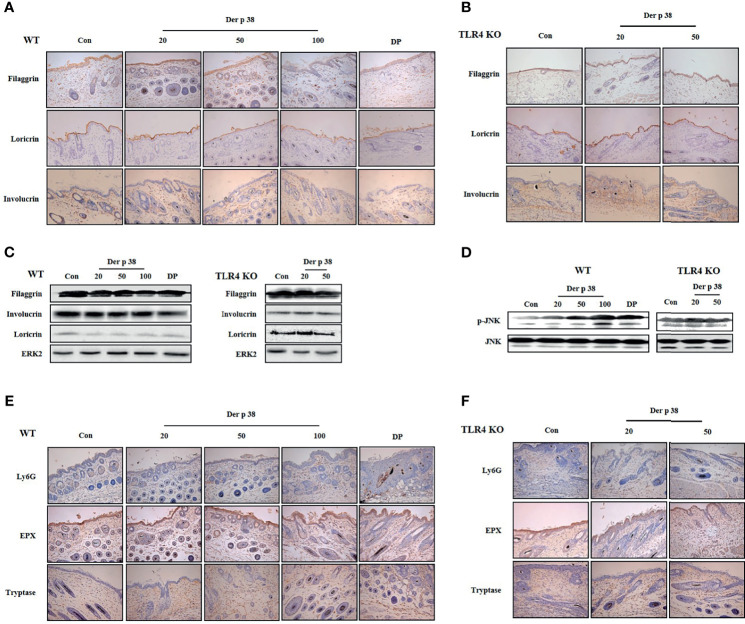
Der p 38 decreases the expression of filaggrin and increases neutrophils, eosinophils and mast cells in the skin of mice. **(A, B, E, F)** For skin barrier proteins and inflammatory cell analysis, skin sections of wild type (WT) **(A, E)** and TLR4 KO mice **(B, F)** were fixed, embedded in paraffin, and incubated with primary antibodies against filaggrin, loricrin, involucrin, Ly6G, eosinophil peroxidase (EPX) and tryptase. The samples were examined by light microscopy (magnification, ×200). Filaggrin, loricrin, involucrin **(C)**, and phospho-JNK **(D)** in tissue lysates were analyzed by Western blotting.

For evaluating the effect of Der p 38 in the pathogenesis of AD, we examined both histological and serological alterations in mice. In WT mice, the skin became thick, and severe erythema, hemorrhage, edema, scarring and erosion were observed after Der p 38 administration; this skin severity was decreased in TLR4 KO mice (**Figure 4A**). Histological features showed severe skin lesions and inflammation, and increased thickness of epidermis and dermis due to Der p 38 (**Figures 4B–D**). Absence of TLR4 decreased these effects of Der p 38. Moreover, total IgE and Der p 38-specific IgE in WT mice was higher than in TLR4 KO mice (**Figures 4E, F**). To investigate alterations of skin barrier proteins and inflammatory cells in detail, we applied immunohistological tools and Western blotting. The expressions of loricrin, involucrin and filaggrin were diminished, whereas JNK was activated in the Der p 38 treated groups (**Figures 5A–D**). As shown in **Figures 5E, F**, neutrophils and eosinophils were infiltrated into the dermis of skin. The number of mast cells was also increased in the Der p 38-treated group. However, Der p 38 had no effect on the skin barrier proteins and inflammatory cells in the TLR4 KO group. These results clearly indicate that Der p 38 is a strong inducer of AD *in vivo*, and TLR4 is the main receptor of Der p 38 (**Figures 2** and **3**).

## Discussion

Although destruction of filaggrin is an essential step in the induction and aggravation of AD, the specific allergen protein that directly regulates filaggrin expression and its mechanism has yet to be determined. As shown in **Figures 2**, **3** and **5**, Der p 38 sensitization reduces the filaggrin expression *in vitro* and *in vivo*. HDM extract induces the downregulation of filaggrin expression. Previous studies reported that the protease allergen assists the entrance of other allergens, and Der p 1 lyses the transmembrane adhesion proteins of tight junctions (28, 29). Sar s 3 is directly capable of cleaving filaggrin (30), and lack of filaggrin increases exposure of Fel d 1 and Der p 1 (31). Although it is known that Der p 38 has a direct cleavage activity on filaggrin, Der p 38 may also facilitate the infiltration of other allergens, thereby resulting in the development and aggravation of allergy by protease activity (**Figure 1D**). Mediators produced by Der p 38 in human keratinocytes inhibit neutrophil apoptosis as well as filaggrin expression (**Figures 3A, D**). The increased expressions of IL-6, IL-8, and MCP-1 by Der p 38 may assist in suppression of neutrophil apoptosis, as determined in our previous paper (15) (**Figure 3B**). Additionally, there was increased infiltration of neutrophils in the skin of the mice (**Figure 5E**). Der p 38 also enhanced the number of eosinophils in the skin. Th2 cytokines such as IL-4, IL-5, and IL-13, and Th2 promoting cytokines such as IL-33, are reported to block filaggrin expression (3, 32). These cytokines function as survival factors of eosinophils by inhibiting apoptosis. However, the Der p 38-treated supernatant exerted no effect on eosinophil apoptosis (**Figure 3D**). Further studies are required to investigate the exact cytokines that downregulate filaggrin expression, and their interaction with eosinophil infiltration.

TLR4 is considered as an important receptor of the Der p 38 inducing AD pathogenic mechanism, although deletion of TLR4 does not block the Der p 38-induced AD severity completely (**Figure 4**). In contrast to our results, the upregulation of TLR4 expression may be related to modulation of AD development as well as protection of infection. Deficiency of TLR4 induces the aggravation of AD after 2,4-dinitrochlorobenzene (DNCB) administration, *via* increased Th2 responses (22). In addition, TLR4 suppresses the pathogenic mechanism of AD induced by *Aspergillus fumigatus* extracts (20). The data of other research groups are not associated with specific allergen proteins. Der p 2 triggers the clinical features of AD *via* TLR2 but not TLR4, despite Der p 2 being an MD2-like protein and inducing asthmatic inflammation through TLR4 (17, 23). In fact, TLR4 expression prevents the Der p 2-induced allergic reactions, thereby questioning the fact that Der p 2 is a TLR4-binding allergen. We think that for the first time, our results demonstrate that the Der p 38 allergen triggers AD and probably worsens the disease *via* TLR4. In addition, LPS suppressed filaggrin expression and induced cytokine secretion like Der p 38 (**Supplementary Figure 3**) (**Figures 2** and **3**). This finding contributes to understanding the role of TLR4 in AD pathogenesis, compared to the current concept of TLR4. Der p 38, the TLR4 binding mite allergen, inhibits filaggrin expression *via* TLR4-related downstream cascade, including PI3K, AKT, JNK and NF-κB pathway (**Figures 2** and **5C, D**). JNK activation is a pivotal signal protein in downregulation of filaggrin expression, but NF-kB is not related to the decreased expression (8). In fact, AKT activation is related to keratinocyte differentiation, and mTORC2 kinase phosphorylates the AKT responsible for filaggrin processing (33, 34). Skin barrier disruption exerts upregulation of pro-inflammatory cytokine expressions in relapsing chronic inflammation, and vice versa. Hence, Der p 38 may aggravate AD symptoms *via* the common pathways (PI3K, AKT, JNK and NF-κB) involved in filaggrin downregulation and cytokine upregulation. Identification of detailed signal mechanism is required to confirm the more precise role of Der p 38 and elucidate an approach of drug development for AD (35, 36).

Neutrophils are the first protectors against infections. Lack of neutrophils causes AD subjects to be very sensitive to the infections, and results in AD exacerbation (37, 38). Der p 2 has no effect on neutrophil infiltration, and addition of LPS to Der p 2 treatment enhances the neutrophil movement (23). Allergens such as Der p 1 and Der p 2 enhance the expressions of T cells, eosinophils and mast cells in the skin (23, 39). Studies have reported the association of neutrophilic inflammation with AD (40, 41). A recent paper has demonstrated that neutrophils are strongly required for the itch that appears during the development of AD. CXCR3-positive sensory neutrons stimulated with CXCL10 are dependent on the itch induced by neutrophils (42). Neutrophilic inflammation, along with mast cells and eosinophils, have been demonstrated in the skin of Der p 38-adminstered mice (**Figures 5E, F**), and the mechanism related to the inflammation was confirmed by discovering the anti-apoptotic signal pathway induced by Der p 38 in human neutrophils (**Figure 3D**). Currently, the role of neutrophils is unknown in AD pathogenesis induced by Der p 38, and this puzzle is targeted to be solved in the near future.

**Figure 6:**
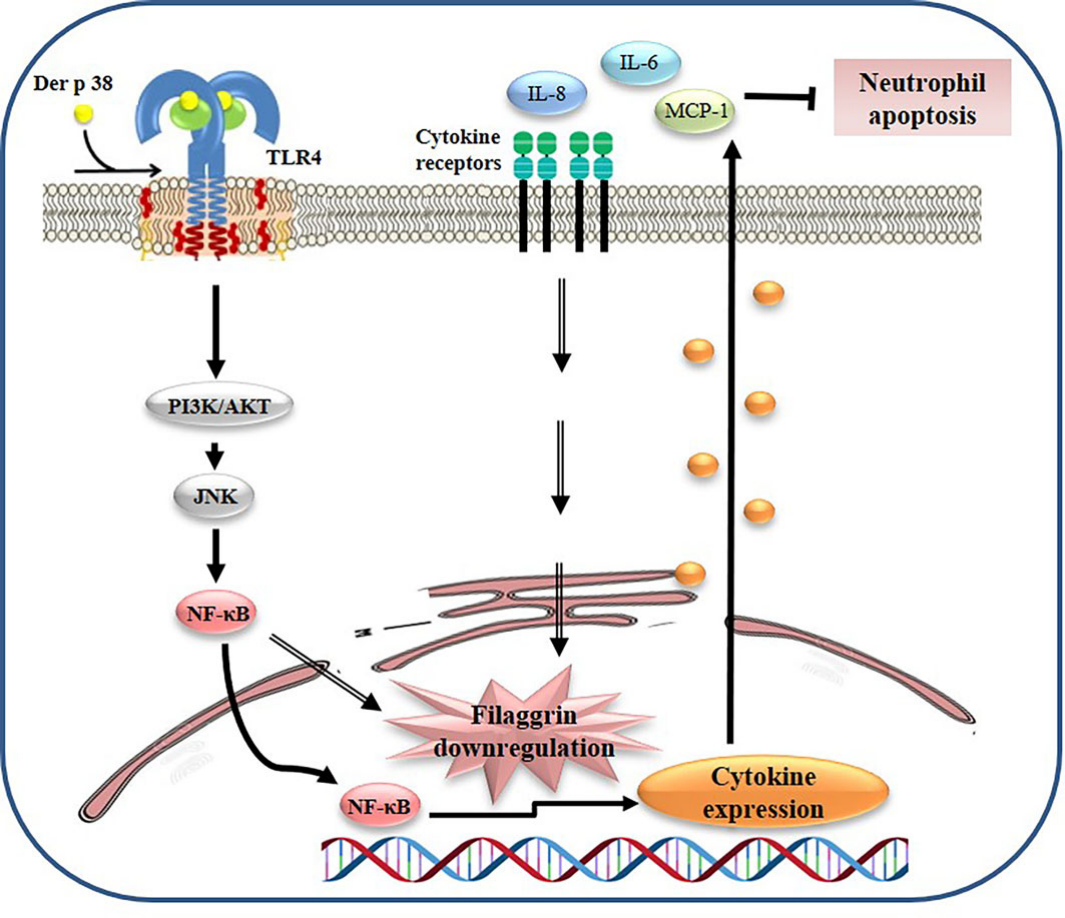
Proposed mechanism of filaggrin downregulation due to Der p 38 in AD. Der p 38 suppresses filaggrin expression and induces cytokine release through TLR4, PI3K, AKT, JNK, and NF-κB, which blocks neutrophil apoptosis.

Our findings have identified the novel allergen, Der p 38, which induces AD pathogenesis by downregulation of filaggrin and upregulation of inflammatory responses such as cytokine secretion and neutrophil survival (**Figure 6**). The pathogenic mechanism of Der p 38 involves signal proteins such as TLR4, PI3K, AKT, JNK, and NF-κB. It would help us to understand the association of HDM with AD, for effectively developing preventive or therapeutic strategies to combat this disease.

## Data Availability Statement

The datasets presented in this study can be found in online repositories. The names of the repository/repositories and accession number(s) can be found in the article/**Supplementary Material**.

## Ethics Statement

The human blood protocol and associated consent forms were reviewed and approved by the Institutional Review Board (IRB) of the Eulji University. The patients/participants provided their written informed consent to participate in this study. All experimental animal procedures were performed in accordance with protocols approved by the Institutional Animal Care and User Committee of Eulji University.

## Author Contributions

HJ and GK designed the research and performed most of the experiments. AK, MH, J-SL, and YH helped conducting the experiments related to animal, and healthy and allergic subjects. BSP analyzed structure of Der p 38. EJY and ISK contributed to the design of the study and supervised the project. All authors contributed to the article and approved the submitted version.

## Funding

This research was supported by National Research Foundation of Korea (NRF) grant funded by the Korea government Ministry of Science and ICT (No. 2017R1C1B507674614).

## Conflict of Interest

The authors declare that the research was conducted in the absence of any commercial or financial relationships that could be construed as a potential conflict of interest.

## Publisher’s Note

All claims expressed in this article are solely those of the authors and do not necessarily represent those of their affiliated organizations, or those of the publisher, the editors and the reviewers. Any product that may be evaluated in this article, or claim that may be made by its manufacturer, is not guaranteed or endorsed by the publisher.

## Supplementary Material

Supplementary Figure 1 Der p 38 directly binds to TLR4. Recombinant Der p 38 protein was added to a TLR4-bound column, eluted, and separated by SDS-PAGE. The gel was stained with silver stain.

Supplementary Figure 2 Der p 38 has no effect on total and surface TLR4 expression in HaCaT cells. **(A, B)** HaCaT cells were treated with 10 μg/mL Der p 38 in the indicated time. The harvested cells were lysed, and TLR4 expression was detected by Western blotting **(A)** and flow cytometry **(B)**.

Supplementary Figure 3 LPS diminishes filaggrin expression and induces cytokine secretion in human keratinocyte HaCaT cells. **(A)** HaCaT cells were treated with 1 μg/mL LPS for the indicated time or 10 μg/mL Der p 38 for 48 h. The harvested cells were lysed, and filaggrin expression was analyzed by Western blotting. **(B)** The cells were treated with 1 μg/mL LPS or 10 μg/mL Der p 38 for 48 h. The supernatant was collected and analyzed by ELISA. The data are presented relative to the control (Con), which was set at 100% as the mean ± S.D. ***p* < 0.01 indicates a significant difference between the untreated and LPS or Der p 38-treated groups,

## References

[1] Weidinger S Novak N. Atopic Dermatitis. Lancet (2016) 387:1109–22. doi: 10.1016/S0140-6736(15)00149-X 26377142

[2] Bieber T. Atopic Dermatitis. N Engl J Med (2008) 358:1483–94. doi: 10.1056/NEJMra074081 18385500

[3] Sullivan M Silverberg NB. Current and Emerging Concepts in Atopic Dermatitis Pathogenesis. Clin Dermatol (2017) 35:349–53. doi: 10.1016/j.clindermatol.2017.03.006 28709564

[4] Weidinger S Beck LA Kabashima K Irvine AD. Atopic Dermatitis. Nat Rev Dis Primers (2018) 4:1–10. doi: 10.1038/s41572-018-0001-z 29930242

[5] Peng W Novak N. Pathogenesis of Atopic Dermatitis. Clin Exp Allergy (2015) 45:566–74. doi: 10.1111/cea.12495 25610977

[6] Lee AY. Molecular Mechanism of Epidermal Barrier Dysfunction as Primary Abnormalities. Int J Mol Sci (2020) 21:1194. doi: 10.3390/ijms21041194 PMC7072774 32054030

[7] Palmer CN Irvine AD Terron-Kwiatkowski A Zhao Y Liao H Lee SP. Common Loss-of-Function Variants of the Epidermal Barrier Protein Filaggrin Are a Major Predisposing Factor for Atopic Dermatitis. Nat Genet (2006) 38:441–46. doi: 10.1038/ng1767 16550169

[8] Kim BE Howell MD Guttman-Yassky E Gilleaudeau PM Cardinale IR Boguniewicz M. TNF-α Downregulates Filaggrin and Loricrin Through C-Jun N-Terminal Kinase: Role for TNF-α Antagonists to Improve Skin Barrier. J Invest Dermatol (2011) 131:1272–79. doi: 10.1038/jid.2011.24 PMC8609659 21346775

[9] Cha KJ Song CS Lee JS Kashif A Hong MH Kim G. Chaenomeles Sinensis Koehne Extract Suppresses the Development of Atopic Dermatitis-Like Lesions by Regulating Cytokine and Filaggrin Expression in NC/Nga Mice. Int J Med Sci (2019) 16:1604–13. doi: 10.7150/ijms.37854 PMC6909810 31839748

[10] Tomas WR Hales BJ Smith WA. House Dust Mite Allergens in Asthma and Allergy. Trends Mol Med (2010) 16:321–28. doi: 10.1016/j.molmed.2010.04.008 20605742

[11] Miller JD. The Role of Dust Mites in Allergy. Clin Rev Allergy Immunol (2019) 57:312–29. doi: 10.5415/apallergy.2013.3.2.79 29936683

[12] Kim KH. Overview of Atopic Dermatitis. Asia Pac Allergy (2013) 3:79–87. doi: 10.5415/apallergy.2013.3.2.79 23667830 PMC3643056

[13] Mueller GA Glesner J Daniel JL Zhang J Hyduke N Richardson CM. Mapping Human Monoclonal IgE Epitopes on the Major Dust Mite Allergen Der P 2. J Immunol (2020) 205:1999–07. doi: 10.4049/jimmunol.2000295 PMC7541722 32907999

[14] Hammad H Chieppa M Perros F Willart MA Germain RN Lambrecht BN. House Dust Mite Allergen Induces Asthma *via* Toll-Like Receptor 4 Triggering of Airway Structural Cells. Nat Med (2009) 15:410–16. doi: 10.1038/nm.1946 PMC2789255 19330007

[15] Kim EH Lee JS Lee NR Baek SY Kim EJ Lee SJ. Regulation of Constitutive Neutrophil Apoptosis Due to House Dust Mite Allergen in Normal and Allergic Rhinitis Subjects. PloS One (2014) 9:e105814. doi: 10.1371/journal.pone.0105814 25243400 PMC4171368

[16] Koller B Müller-Wiefel AS Rupec R Korting HC Ruzicka T. Chitin Modulates Innate Immune Responses of Keratinocytes. PloS One (2011) 6:e16594. doi: 10.1371/journal.pone.0016594 21383982 PMC3044707

[17] Trompette A Divanovic S Visintin A Blanchard C Hegde RS Madan R. Allergenicity Resulting From Functional Mimicry of a Toll-Like Receptor Complex Protein. Nature (2009) 457:585–88. doi: 10.1038/nature07548 PMC2843411 19060881

[18] Mandron M Ariès MF Boralevi F Martin H Charveron M Taieb A. Age-Related Differences in Sensitivity of Peripheral Blood Monocytes to Lipopolysaccharide and Staphylococcus Aureus Toxin B in Atopic Dermatitis. J Invest Dermatol (2008) 128:882–89. doi: 10.1038/sj.jid.5701112 17960185

[19] Panzer R Blobel C Fölster-Holst R Proksch E. TLR2 and TLR4 Expression in Atopic Dermatitis, Contact Dermatitis and Psoriasis. Exp Dermatol (2014) 23:364–66. doi: 10.1111/exd.12383 24661005

[20] Brandt EB Gibson AM Bass S Rydyznski C Khurana Hershey GK. Exacerbation of Allergen-Induced Eczema in TLR4- and TRIF-Deficient Mice. J Immunol (2013) 191:3519–25. doi: 10.4049/jimmunol.1300789 PMC3788607 23997219

[21] Tao Y Wang Y Wang X Wang C Bao K Ji L. Calycosin Suppresses Epithelial Derived Initiative Key Factors and Maintains Epithelial Barrier in Allergic Inflammation *via* TLR4-Mediated NF-κb Pathway. Cell Physiol Biochem (2017) 44:1106–19. doi: 10.1159/000485416 29179209

[22] Lin L Xie M Chen X Yu Y Liu Y Lei K. Toll-Like Receptor 4 Attenuates a Murine Model of Atopic Dermatitis Through Inhibition of Langerin-Positive DCs Migration. Exp Dermatol (2018) 27:1015–22. doi: 10.1111/exd.13698 29851146

[23] Stremnitzer C Jensen-Jarolim E. Epicutaneously Applied Der P 2 Induces a Strong T_H_2-Biased Antibody Response in C57BL/6 Mice, Independent of Functional TLR4. Allergy (2014) 69:741–51. doi: 10.1111/all.12399 PMC4023119 24735481

[24] Lee JS Lee NR Kahif A Yang SJ Nam AR Song IC. S100A8 and S100A9 Promote Apoptosis of Chronic Eosinophilic Leukemia Cells. Front Immunol (2020) 11:1258. doi: 10.3389/fimmu.2020.01258 32903598 PMC7438788

[25] Weghofer M Grote M Resch Y Casset A Kneidinger M Kopec J. Identification of Der P 23, A Peritrophin-Like Protein, as a New Major *Dermatophagoides Pteronyssinus* Allergen Associated With the Peritrophic Matrix of Mite Fecal Pellets. J Immunol (2013) 190:3059–67. doi: 10.4049/jimmunol.1202288 PMC4582595 23460742

[26] Asturias JA Arilla MC Gómez-Bayón N Martínez A Martínez J Palacios R. Sequencing and High Level Expression in *Escherichia Coli* of the Tropomyosin Allergen (Der P 10) From *Dermatophagoides Pteronyssinus* . Biochim Biophys Acta (1998) 1397:27–30. doi: 10.1016/s0167-4781(98)00006-2 9545522

[27] Kim DH Gu A Lee JS Yang EJ Kashif A Hong MH. Suppressive Effects of S100A8 and S100A9 on Neutrophil Apoptosis by Cytokine Release of Human Bronchial Epithelial Cells in Asthma. Int J Med Sci (2020) 17:498–09. doi: 10.7150/ijms.37833 PMC7053304 32174780

[28] Zhang J Chen J Robinson C. Cellular and Molecular Events in the Airway Epithelium Defining the Interaction Between House Dust Mite Group 1 Allergens and Innate Defences. Int J Mol Sci (2018) 19:3549. doi: 10.3390/ijms19113549 PMC6274810 30423826

[29] Jacquet A Robinson C. Proteolytic, Lipidergic and Polysaccharide Molecular Recognition Shape Innate Responses to House Dust Mite Allergens. Allergy (2020) 75:33–53. doi: 10.1111/all.13940 31166610

[30] Beckham SA Boyd SE Reynolds S Willis C Johnstone M Mika A. Characterization of a Serine Protease Homologous to House Dust Mite Group 3 Allergens From the Scabies Mite Sarcoptes Scabiei. J Biol Chem (2009) 284:34413–22. doi: 10.1074/jbc.M109.061911 PMC2797209 19812030

[31] Simpson A Brough HA Haider S Belgrave D Murray CS Custovic A. Early-Life Inhalant Allergen Exposure, Filaggrin Genotype, and the Development of Sensitization From Infancy to Adolescence. J Allergy Clin Immunol (2020) 145:993–01. doi: 10.1016/j.jaci.2019.08.041 PMC7057264 31629803

[32] Imai Y. Interleukin-33 in Atopic Dermatitis. J Dermatol Sci (2019) 96:2–7. doi: 10.1016/j.jdermsci.2019.08.006 31455506

[33] Naeem AS Zhu Y Di WL Marmiroli S O’Shaughnessy RF. AKT1-Mediated Lamin a/C Degradation Is Required for Nuclear Degradation and Normal Epidermal Terminal Differentiation. Cell Death Differ (2015) 22:2123–32. doi: 10.1038/cdd.2015.62 PMC4816115 26045045

[34] Ding X Willenborg S Bloch W Wickström SA Wagle P Brodesser S. Epidermal Mammalian Target of Rapamycin Complex 2 Controls Lipid Synthesis and Filaggrin Processing in Epidermal Barrier Formation. J Allergy Clin Immunol (2020) 145:283–00. doi: 10.1016/j.jaci.2019.07.033 31401286

[35] Amano W Nakajima S Kunugi H Numata Y Kitoh A Egawa G. The Janus Kinase Inhibitor JTE-052 Improves Skin Barrier Function Through Suppressing Signal Transducer and Activator of Transcription 3 Signaling. J Allergy Clin Immunol (2015) 136:667–77. doi: 10.1016/j.jaci.2015.03.051 26115905

[36] Kusari A Han AM Schairer D Eichenfield LF. Atopic Dermatitis: New Developments. Dermatol Clin (2019) 37:11–20. doi: 10.1016/j.det.2018.07.003 30466683

[37] De Benedetto A Agnihothri R McGirt LY Bankova LG Beck LA. Atopic Dermatitis: A Disease Caused by Innate Immune Defects? J Invest Dermatol (2009) 129:14–30. doi: 10.1038/jid.2008.259 19078985

[38] Werfel T. The Role of Leukocytes, Keratinocytes, and Allergen-Specific IgE in the Development of Atopic Dermatitis. J Invest Dermatol (2009) 129:1878–91. doi: 10.1038/jid.2009.71 19357709

[39] Szalai K Kopp T Lukschal A Stremnitzer C Wallmann J Starkl P. Establishing an Allergic Eczema Model Employing Recombinant House Dust Mite Allergens Der P 1 and Der P 2 in BALB/c Mice. Exp Dermatol (2012) 21:842–46. doi: 10.1111/exd.12015 PMC3532600 23163649

[40] Koro O Furutani K Hide M Yamada S Yamamoto S. Chemical Mediators in Atopic Dermatitis: Involvement of Leukotriene B4 Released by a Type I Allergic Reaction in the Pathogenesis of Atopic Dermatitis. J Allergy Clin Immunol (1999) 103:663–70. doi: 10.1016/s0091-6749(99)70240-x 10200017

[41] Choy DF Hsu DK Seshasayee D Fung MA Modrusan Z Martin F. Comparative Transcriptomic Analyses of Atopic Dermatitis and Psoriasis Reveal Shared Neutrophilic Inflammation. J Allergy Clin Immunol (2012) 130:1335–43. doi: 10.1016/j.jaci.2012.06.044 PMC3511596 22920495

[42] Walsh CM Hill RZ Schwendinger-Schreck J Deguine J Brock EC Kucirek N. Neutrophils Promote CXCR3-Dependent Itch in the Development of Atopic Dermatitis. Elife (2019) 8:e48448. doi: 10.7554/eLife.48448 31631836 PMC6884397

