# Supplementary material for: Pathogenic Mechanism of Der p 38 as a Novel Allergen Homologous to RipA and RipB Proteins in Atopic Dermatitis
Source: Front Immunol. 2021 Oct 8;12:646316. doi: 10.3389/fimmu.2021.646316 (PMC8531521; doi:10.3389/fimmu.2021.646316)
Supplement: Supplementary Figure 1 — Der p 38 directly binds to TLR4. Recombinant Der p 38 protein was added to a TLR4-bound column, eluted, and separated by SDS-PAGE. The gel was stained with silver stain. [file Presentation_1.ppt]

## Slide 1
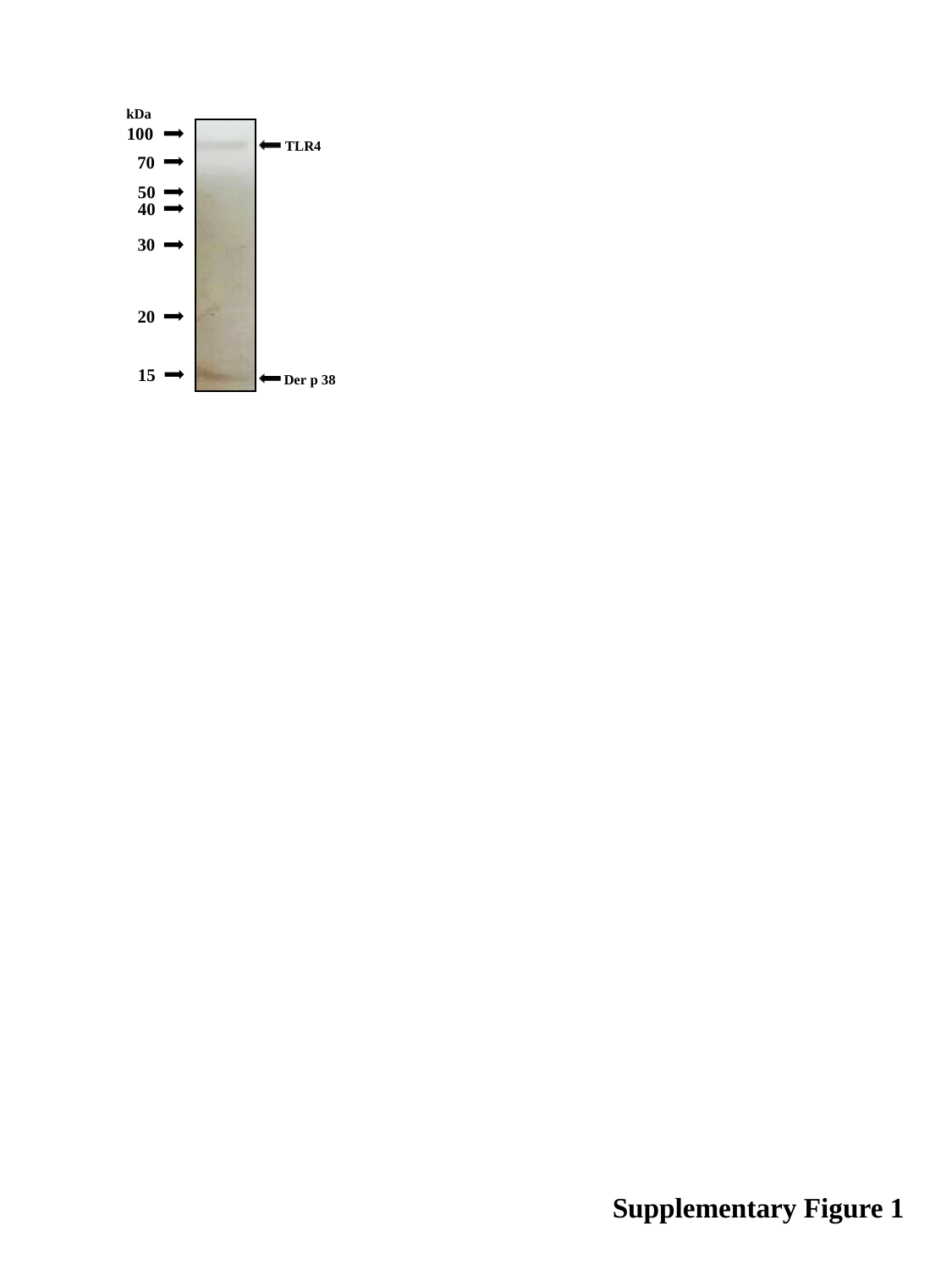

kDa
100
TLR4
70
50
40
30
20
15
Der p 38
Supplementary Figure 1

## Slide 2
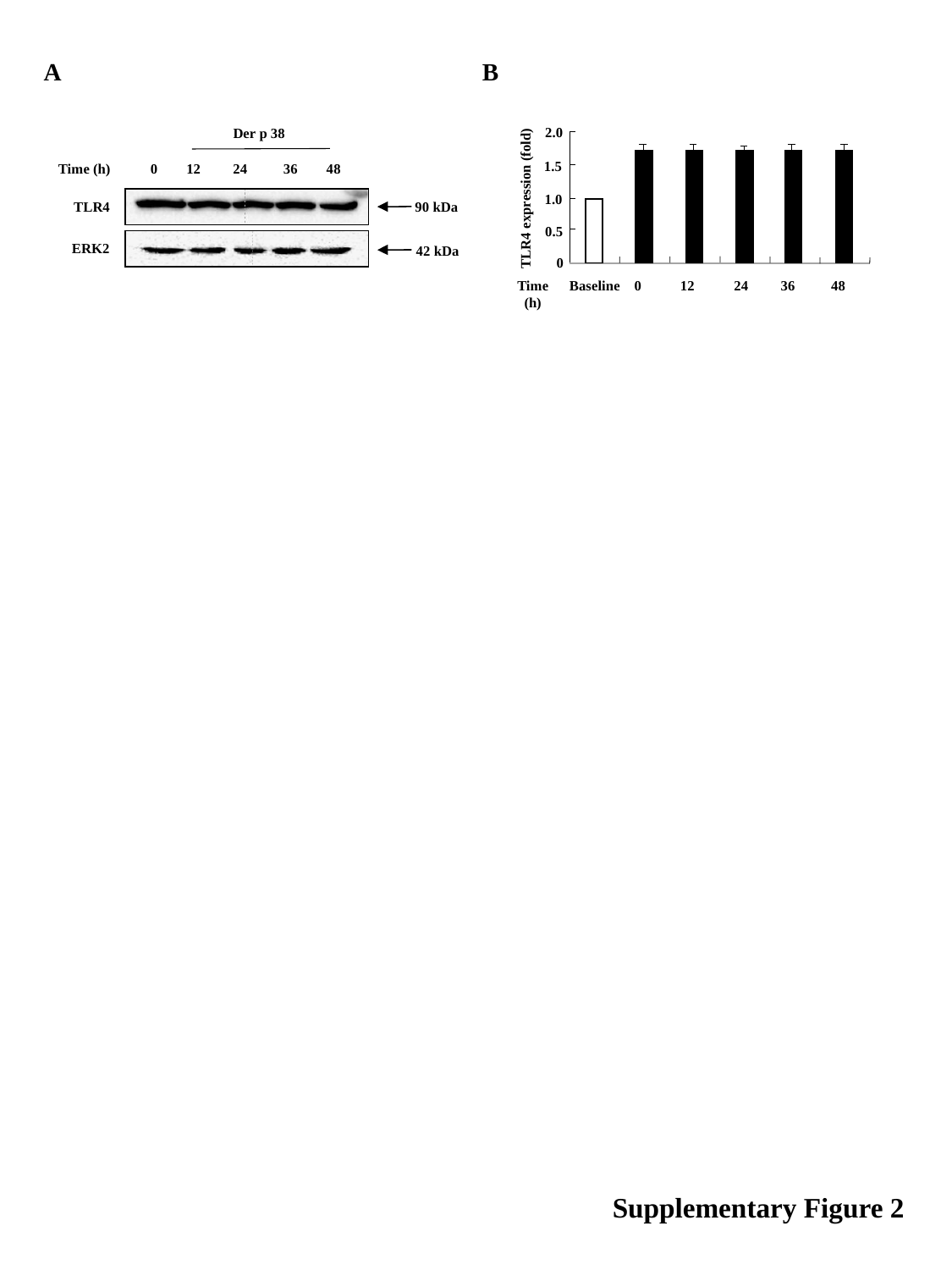

B
A
Der p 38
2.0
Time (h)
 0 12 24 36 48
1.5
TLR4 expression (fold)
1.0
TLR4
90 kDa
0.5
ERK2
ERK2
42 kDa
0
Time (h)
Baseline
0
12
24
36
48
Supplementary Figure 2

## Slide 3
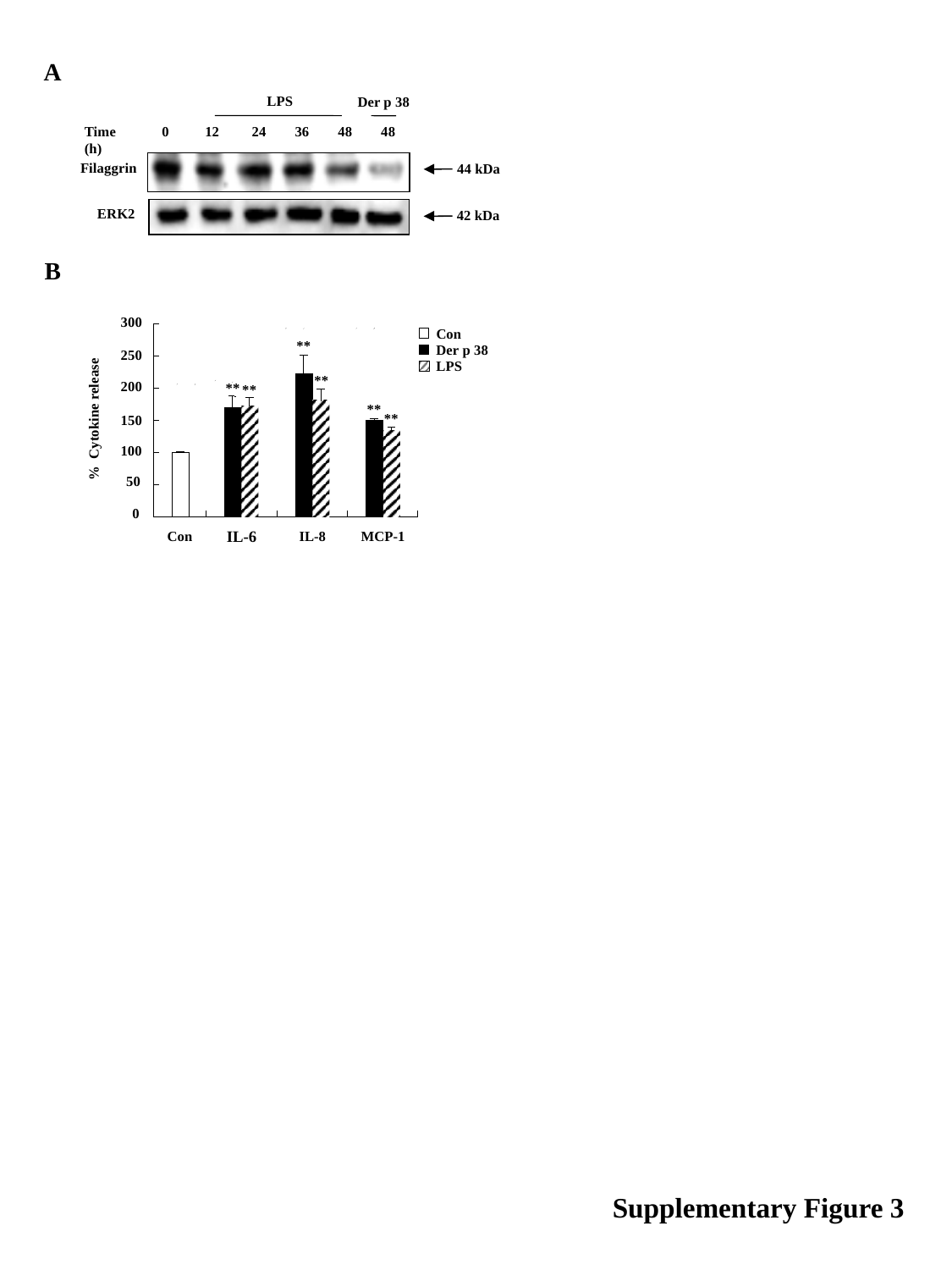

A
LPS
Der p 38
Time (h)
 0 12 24 36 48 48
Filaggrin
44 kDa
ERK2
42 kDa
B
300
Con
**
Der p 38
250
LPS
**
**
**
200
**
% Cytokine release
**
150
100
50
0
IL-6
Con
IL-8
MCP-1
Supplementary Figure 3
