# Supplementary material for: Pathogenic Mechanism of Der p 38 as a Novel Allergen Homologous to RipA and RipB Proteins in Atopic Dermatitis
Source: Front Immunol. 2021 Oct 8;12:646316. doi: 10.3389/fimmu.2021.646316 (PMC8531521; doi:10.3389/fimmu.2021.646316)
Supplement: Supplementary file 2 [file Table_1.docx]

**Supplementary Table 1. The numbers of peripheral blood cells of normal and AD subjects**

|  | Normal (n=19) | AD (n=19) |
| --- | --- | --- |
| RBC (10^6^/μL) | 4.6 ± 0.2 (4.1~4.9) | 4.7 ± 0.2 (4.3~5.0) |
| Platelet (10^3^/μL) | 292 ± 57 (203~387) | 321.3 ± 66.3 (225~479) |
| WBC (10^3^/μL) | 7.2 ± 1.9 (4.8~11.5) | 6.5 ± 1.0 (8.7~4.9) |
| Neutrophil (%) | 60.9 ± 5.2 (52.1~66.6) | 56.5 ± 6.2 (63.9~49.2) |
| Lymphocyte (%) | 30.4 ± 5.0 (25.3~39.7) | 34.1 ± 5.7 27.2~41.6) |
| Monocyte (%) | 6.5 ± 1.1 (5.0~8.0) | 6.8 ± 1.5 (5.4~10) |
| Eosinophil (%) | 1.6 ± 0.6 (0.5~2.4) | 2.1 ± 0.9 (0.6~3.3) |
| Basophil (%) | 0.6 ± 0.4 (0.3~1.2) | 0.5 ± 0.2 (0.1~0.9) |

AD, atopic dermatitis
